# Optical and Electronic Properties of Organic NIR-II Fluorophores by Time-Dependent Density Functional Theory and Many-Body Perturbation Theory: *GW*-BSE Approaches

**DOI:** 10.3390/nano11092293

**Published:** 2021-09-03

**Authors:** Nguyet N. T. Pham, Seong Hun Han, Jong S. Park, Seung Geol Lee

**Affiliations:** 1 School of Chemical Engineering, Pusan National University, Busan 46241, Korea; 201799128@pusan.ac.kr (N.N.T.P.); eggmoney8759@pusan.ac.kr (S.H.H.); 2 Department of Organic Material Science and Engineering, Pusan National University, Busan 46241, Korea

**Keywords:** organic fluorescent dyes, NIR, time-dependent density functional theory (TDDFT), many-body perturbation theory (MBPT), *GW*-BSE, optical property, electronic property

## Abstract

Organic-molecule fluorophores with emission wavelengths in the second near-infrared window (NIR-II, 1000–1700 nm) have attracted substantial attention in the life sciences and in biomedical applications because of their excellent resolution and sensitivity. However, adequate theoretical levels to provide efficient and accurate estimations of the optical and electronic properties of organic NIR-II fluorophores are lacking. The standard approach for these calculations has been time-dependent density functional theory (TDDFT). However, the size and large excitonic energies of these compounds pose challenges with respect to computational cost and time. In this study, we used the *GW* approximation combined with the Bethe-Salpeter equation (*GW*-BSE) implemented in many-body perturbation theory approaches based on density functional theory. This method was used to perform calculations of the excited states of two NIR molecular fluorophores (BTC980 and BTC1070), going beyond TDDFT. In this study, the optical absorption spectra and frontier molecular orbitals of these compounds were compared using TDDFT and *GW*-BSE calculations. The *GW*-BSE estimates showed excellent agreement with previously reported experimental results.

## 1. Introduction

Organic fluorescent dyes have received intense attention because of their high fluorescence quantum efficiency, easy synthesis, and adjustable luminescence spectrum [1,2,3,4,5,6,7]. Recently, fluorescence imaging in the second near-infrared window (NIR-II, 1000–1700 nm) has shown strong potential in biological tissues compared with fluorescence imaging in the ultraviolet (UV), visible (400–700 nm), and first near-infrared window (NIR-I, 700–1000 nm) regions because of reduced scattering of photons and self-luminescence [8,9,10,11,12,13,14]. NIR-II fluorophores are mainly based on two architectures that extend the absorption emission wavelength range: donor-acceptor-donor (DAD) fluorophores and polymethine cyanines [15,16]. Several commercial NIR-II fluorophores, including IR1048 [9,17] and IR1061 [9,17,18], exhibit ideal fluorescence in the wavelength region beyond 1000 nm. Adjusting the optical properties into the NIR-II range while maintaining a favorable antisolvent quenching ability remains a substantial challenge [19]. The wavelength of cyanine fluorophores can be tuned by installing terminal benzothiopyrylium heterocycles at different positions of the electron diethylamino particles. Notably, the benzothiopyrylium pentamethine cyanines (BTCs) overcome the phenomenon of solvatochromism in polar solvents, which is the key to obtaining various anti-quenching dyes with high brightness and superior photostability in aqueous solution. Wang et al. [19] demonstrated that BTC1070 predicts superior photostability, which will benefit long-time observation, especially for imaging-guided surgical operations. The dyes’ emission frequency can be tuned toward deep blue. Furthermore, the synthesis of heteroleptic complexes by replacing one ligand in a homoleptic compound with another can improve the emitter performance and stability and modify the emission frequency to achieve the desired color [20,21].

For new efficient fluorophores to be designed, a theoretical basis is needed to understand and predict their measured photophysical and electronic properties that result from extending their conjugated chains and adjusting their terminal groups. Among the available methods, time-dependent density functional theory (TDDFT) such as B3LYP is widely used to simulate excited states because of its moderate computing requirements [22,23,24,25,26,27,28]. In this research, we looked for the state-of-the-art effective computational method to accurately excited states for cyanine NIR fluorophores systems, constituting a chain containing sp^2^-hybridized carbon atoms forming a conjugated bond. TDDFT calculations based on local-density approximations (LDA) with exchange-correlation (XC) provide suitable excitation energies for small and medium-sized molecules. Stephan Kümmel [29] showed that tuned range-separated hybrid functionals could overcome the charge-transfer problem, but it still needs to be adjusted according to a specific compound to have good accuracy.

Although long-range corrected (LRC) TDDFT is more consistent and favorable than traditional hybrids, it still practically depends on an HF/GGA error cancelation [30]. The TDDFT has recently been used through many-body perturbation theory (MBPT) based on Green’s functions within the *GW* approximation by solving the Bethe-Salpeter equation (*GW*-BSE) [30,31,32,33,34]. *GW*-BSE can be applied with the Tamm-Dancoff approximation (TDA), which describes the coupling between resonant and antiresonant electron-hole pairs to reduce the computational cost.

**Figure 1:**
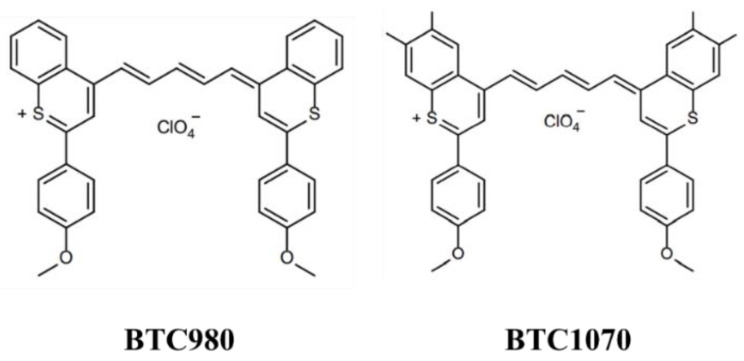
Chemical structures of the BTC980 and BTC1070 fluorophores.

Recently, *GW*-BSE has emerged as a method that can be efficient and accurate for molecules with similar or even reduced errors compared to TDDFT [30,35]. Relative to standard contemporary TDDFT approaches, the BSE method receives a lot of attention from the research community because of its accurate description for charge transfer in molecules’ donor-acceptor complexes and dimers [36,37]. Moreover, the *GW*/BSE approach has been successfully applied to determine excited states in crystals [38,39,40,41,42], polymers [43], and organic molecules [43,44,45,46,47,48,49,50,51]. In particular, BSE formalism can describe charge transfer efficiently and accurately [52,53], especially for cyanine compounds [54]. Furthermore, the new long-range corrected hybrid function, named CAM-B3LYP [55], shows excellent results related to describing excited state properties in large aromatic molecules [56,57], which is poorly shown in LDA, GGA, and hybrid functionals [58]. Therefore, in this study, we simulated the excited states of two different NIR-II fluorophores: BTC980 and BTC1070, as shown in Figure 1. We applied the *GW*-BSE, standard TDDFT (B3LYP), and CAM-B3LYP to investigate the optical and electronic properties of organic NIR-II fluorophores as well as the solvation effect with dichloromethane (DCM).

## 2. Computational Methods

### 2.1. GW-BSE Calculations

We carried out a three-step process to study the electronic and optical excitation using the many-body perturbation theory (MBPT) [59] approach within the *GW* approximation at the Perdew-Burke-Ernzerhof (PBE) [60] exchange-correlation functional. The core of this theory is given by elucidating the two-particle Green’s function and solving its equation of motion using the BSE. Only the gamma point was applied for all calculations.

As the starting point, the ground-state calculation of structures was carried out by applying density functional theory (DFT) [61] in the local density approximation (LDA) and generalized gradient approximation (GGA) implemented in VASP version 5.3.5 (VASP Software GmbH, Vienna, Austria) [62]. The cutoff for the kinetic energy was 450 eV to ensure a total energy convergence better than 10^−4^ eV, applied with only the gamma point for all calculations. Periodic boundary condition (PBC) and supercell techniques were applied with a vacuum of 10 Å along the z-axis to avoid interactions between neighboring images. Norm-conserving pseudopotentials [63,64] were applied for first-row elements, which probably helped by error compensation, to obtain a non-metallic ground state with the PBE calculation. Booth et al. [65] concluded the easy switch between the local-atom-centered Gaussian and periodic plane-wave basis sets, potentially leading to novel, transferable, and more compact basis sets to reduce the computational cost of correlated wave-function-based theories in periodic systems even further. Bachelet et al. [66] concluded that norm-conserving pseudopotentials combined with a reasonably sized Gaussian basis could successfully be used to calculate structural properties self-consistently. Gaussian orbital functions [67] were applied through the Gaussian smearing method (ISMEAR = 0) with a small sigma value (SIGMA = 0.01).

Then, in this simplified ev *GW* scheme [68,69] within the *GW* approximation of many-body Green’s functions theory, as introduced by Hedin and Lundqvist [70], a 50 eV *GW* energy cutoff was applied for all structures and 144 and 240 bands of BTC980 and BTC1070, respectively. Both the Green’s function (G) and the dielectric screening (W) can be obtained from the basis of ground-state exchange–correlate Kohn–Sham functions and energies. The *GW*-BSE method was implemented into our original program code using the base method of all electrons [51,71,72,73,74]. Eighty frequency points (NONMEGA = 80), 8 empty bands, and 10^−6^ eV of energy convergence along with 10 occupied and 15 unoccupied bands were applied in the calculations. The final goal of the BSE was solved by diagonalizing the well-known electron–hole pair Hamiltonian [75] with the scheme of the beyond Tamm-Dancoff approximation [51] by including resonant-antiresonant transition coupling corresponding to the setting of ANTIRES = 2 to get the absorption spectra. The poles of the Green’s function then determined the QP energies.

The emission calculations were reserved for the absorption process. The emission spectra showed a Stokes shift with adsorption, which was due to the structure relaxation after creating an electron-hole pair. The relaxed atomic positions corresponding to the hole in the HOMO and electron in the LUMO found in the excited state made the electron-hole pair. Then, solving with the Bethe-Salpeter equation (BSE), the imaginary part of dielectric function could also be used to get the emission wavelength since the emission was as the time reversal of adsorption [72,73]. To account for solvation, the *GW* and BSE approach was performed with the state-specific non-equilibrium polarizable continuum model (PCM) [74,75].

In this study, VASPsol [76] was employed with the implicit solvation model using a dielectric constant (ɛ = 8.93) for dichloromethane (DCM) for the calculations including the solvation effect. For implicit solvation, the polarizable continuum model (PCM) was optimized using the equilibrium regime within the linear response (LR)-PCM approach. Lee [77] predicted the combination of *GW* formalism with the equilibrium PCM in a plane-wave basic set within local approximation. Azarias et al. [78] also concluded that the BSE/ev *GW* is the most accurate approach for LR-PCM solvent. So, at the last step, the equilibrium LR-PCM was continuously solved with Bethe–Salpeter equation for the excited-state calculation of our BTC980 and BTC1070 dyes in DCM medium.

### 2.2. TDDFT Calculations

The standard and long-range corrected hybrid function was figured out by applying it in the Gaussian16 package [79], named B3LYP and CAM-B3LYP, respectively. The optimized geometries of two complexes in their electronic ground states were obtained from B3LYP using a 6-311G (d, p) basic set. All the optimizations were done without constraint on dihedral angles. The predicted energy of the excited state for electronic and optical properties was investigated using TD-DFT/CAM-B3LYP and TDDFT/B3LYP calculations in neutral on the fully DFT-optimized geometries. The dichloromethane (DCM) solvent was applied for linear response (LR) in the non-equilibrium solvation polarizable continuum model (PCM). All geometries were visualized using either the GaussView 6.1.1 (Semichem Inc., Shawnee Mission, KS, USA) [80] or Gabedit 2.5.0 [81] software package.

## 3. Results and Discussion

### 3.1. Optical Property

In this research, we compared how reasonable the match between theory and experimental values was through the vertical absorption wavelength, which only requires the optimization of the ground-state (GS) structure, whereas the vertical absorption energy ($E^{vert-abs}$) can be computed with Equation (1) [82]:

(1) $$E^{vert-abs}=E^{ES}-E^{GS}$$

where $E^{ES}$ and $E^{GS}$ describe the excited state and ground-state energies, respectively. The computed vibronic spectra based on the coupling between the vibrational-electronic can be expected to give accuracy in $E_{abs}=\frac{hc}{\lambda_{max}}$. In addition, the Herzberg-Teller vibronic coupling effects of the large molecules can be neglected [83]. Therefore, the experimental spectra can be converted into $E_{abs}=\frac{hc}{\lambda_{max}}$ with high accuracy through the Duschinsky effects [84] within the Franck-Condon approximation [85,86].

**Figure 2:**
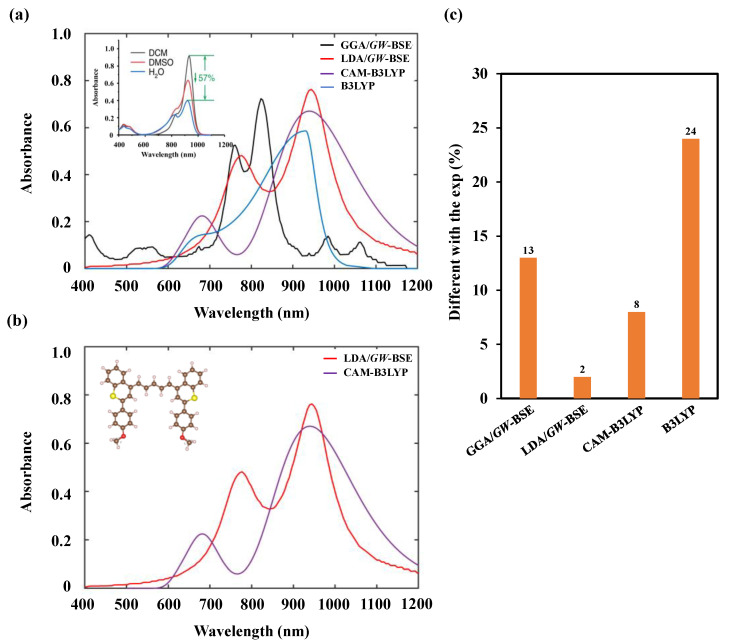
The UV/VIS absorption spectra of BTC980 under (**a**) *GW*-BSE within the VASP package, and the standard hybrid (B3LYP) and long-range corrected hybrid (CAM-B3LYP) within the Gaussian package. (**b**) The best absorption spectra match the experiment. (**c**) The difference value of the vertical theoretical absorption wavelength ($\lambda_{vert-abs}$ ) and the experimental wavelength ($\lambda_{exp-abs})$, $\Delta_{\lambda }\left(nm\right)=\lambda_{exp-abs}-\lambda_{vert-abs}$. The inset in Figure 2a: the absorption spectra of BTC980 from the experiment [19].

In Figure 2, we plotted the difference between our vertical theoretical absorption wavelength in various simulation methods and experiments [19] of BTC980 to highlight the qualitative difference between *GW*-BSE and TDDFT. The GGA/*GW*-BSE showed a high vertical absorption wavelength gap value compared to the experimental longest absorption wavelength with 13 nm. By contrast, LDA/*GW*-BSE showed the lowest vertical absorption wavelength gap value of 2 nm compared to the experimental value. The mean-field LDA appeared to be a better starting point than the GGA for *GW*-BSE calculations, not only in terms of the peak position of wavelength but also the sharp of the absorption spectrum. Regarding the TDDFT, CAM-B3LYP predicted the reasonable UV spectra with an 8 nm difference between vertical absorption wavelength compared to the experimental wavelength, whereas that value was 24 nm in the B3LYP result.

**Figure 3:**
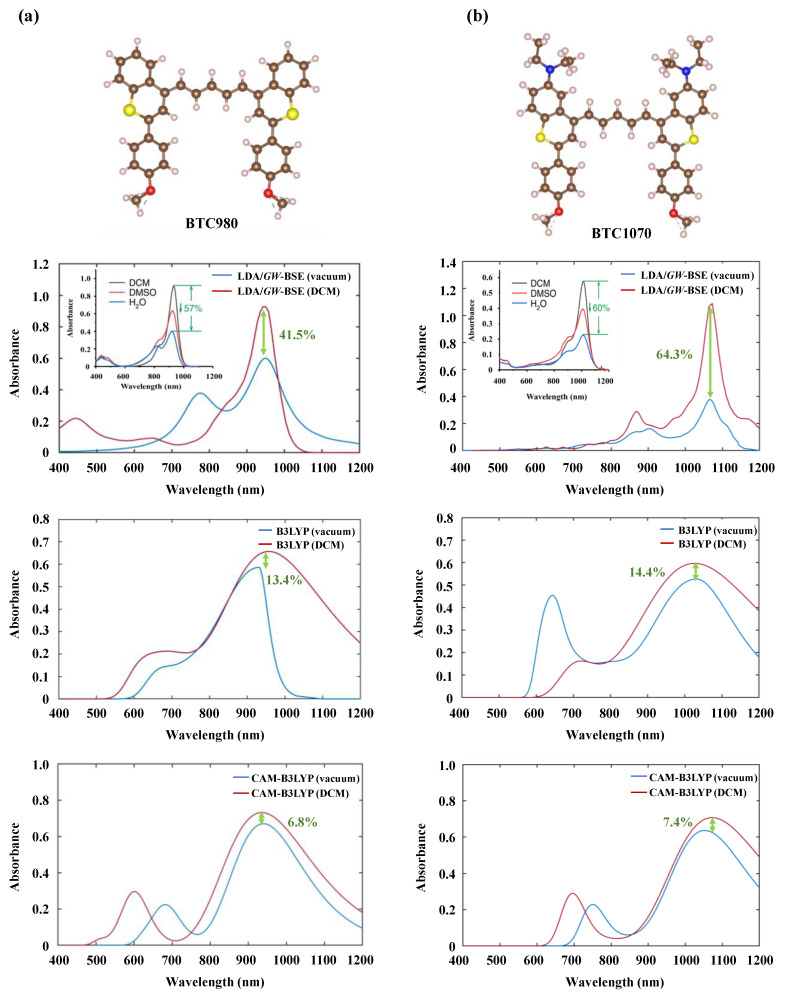
The UV-VIS absorption spectra of (**a**) BTC980 and (**b**) BTC1070 were simulated under *GW*-BSE, B3LYP, and CAM-B3LYP in vacuum and dichloromethane (DCM) solution. The inset figure is the UV-VIS spectra from the experiment [19].

Figure 3 shows the optical absorption spectra of structures based on the LDA/*GW*-BSE, B3LYP, and CAM-B3LYP in both a vacuum and DCM solution compared to those experimentally [19] obtained. Our *GW*-BSE-simulated UV-vis spectra were generally in excellent agreement with the experimental spectra [19], especially with respect to peak positions and peak heights, even in the case of sharp increases in absorption. The agreement was especially strong in the case of the BTC1070 absorption spectra.

The spectral shapes of the simulated (B3LYP) and experimental [19] spectra of BTC980 were similar in the NIR-I region (700–1000 nm), where the simulated spectrum showed the longest absorption/emission peaks at 921.8/931.1 nm. However, the *GW*-BSE calculation showed the longest absorption/emission wavelength at 923.4/975.1 nm, which was closer to the wavelength of the experimentally observed peak at 932/980 nm [19].

The *GW*-BSE optical spectra of BTC1070 showed distinct absorption bands in the visible (400–700 nm), NIR-I (700–1000 nm), and NIR-II (≥1000 nm) regions similar to those in the experimental spectra [18,19,87,88]. Table 1 shows the computed optical and electronic parameters of structures in *GW*-BSE, B3LYP, CAM-B3LYP, and the experimental data. The B3LYP fluorescent emission showed maximum emission wavelengths (λ_fl.max_) that differed and red-shifted from the experimental by approximately 48.9 nm (BTC980) [19] and 47.2 nm (BTC1070) [19]. Meanwhile, the CAM-B3LYP showed that higher the longest emission wavelength from the experiment by 17 nm (BTC980) and lower the longest emission wavelength from the experiment by 4.8 nm (BTC1070), respectively. The *GW*-BSE calculations showed excellent agreement with the most intense experimental maximum absorption/emission wavelengths at 923.4/975.1 nm for BTC980 and 1014.7/1065.4 nm for BTC1070.

### 3.2. Solvation Effect

Considering the solvent influence, we investigated the optical spectrum spectra of BTC980 by using LDA/*GW*-BSE within VASP code and B3LYP and CAM-B3LYP as implemented in the Gaussian package in a vacuum and dichloromethane (DCM) solution. Figure 3 shows the UV-VIS spectra in a vacuum and DCM solution of both BTC980 and BTC1070. The B3LYP absorption spectra show the maximum absorption wavelength of BTC980 that differed and blue-shifted from the experimental absorption wavelength by approximately 15 nm after applying DCM solution. According to Figure 3a, for BTC980 dye in a vacuum, the LDA/*GW*-BSE absorption spectra showed a peak at the NIR-I region (750–900 nm), whereas the CAM-B3LYP and B3LYP spectra showed another peak at the visible region (380–780 nm). Moreover, in terms of the DCM solvent effect, LDA/*GW*-BSE spectra showed that the absorbance intensity of BTC980 and BTC1070 structures increased 41.5% and 64.3%, respectively, compared to without solvent. It can be seen that for the LDA/*GW*-BSE absorption spectra, two peaks appeared at around 650 and 450 nm in the UV-visible region, which almost matched with the absorption spectra in the experiment [19]. The LDA/*GW*-BSE absorption spectra of BTC1070 in the vacuum showed absorption peaks at 1014.7 nm in the NIR-II region and at around 880 nm in the NIR-I region, whereas they appeared at around 1013.5 and 900 nm in the DCM medium. However, the highest wavelength values of BTC980 for *GW*-BSE and CAM-B3LYP were almost similar to the wavelength peak in the gas phase, whereas the absorbance intensity increased 41.5% and 6.8% in the DCM medium, respectively.

The CAM-B3LYP spectra in both BTC980 and BTC1070 showed that the absorption peaks in the visible region blue-shifted in DCM solvent from the neutral environment by approximately 95 and 55 nm, respectively. On the other hand, the B3LYP spectra of BTC1070 illustrated almost a similar highest absorption peak at 1008.7 nm in both neutral and DCM solvent, and the remaining one was around 670 and 715 nm without and with DCM, respectively. The longest absorption wavelength of BTC1070 red-shifted in DCM compared to that in the vacuum by approximately 15 nm. As shown in Figure 3, in our LDA/*GW*-BSE in DCM medium, the absorption spectra of both BTC980 and BTC1070 molecules were in excellent agreement with the experimental spectra [19], especially wavelength peak, absorbance peak, and even the sharp of ranges.

### 3.3. Electronic Property

The frontier excitation energy was evaluated as the energy gap between the highest occupied molecular orbitals (HOMOs) and lowest unoccupied molecular orbitals (LUMO). According to the experimental spectra, the optical bandgap is the excitation energy gap from the ground state (S_0_) to the lowest singlet excited state (S_1_), which is only possible with a highly excited state configuration. The transition energy from the ground state to the excited state corresponds to the HOMO and LUMO. The optical bandgap is approximately equal to the HOMO-LUMO energy gap. Most currently reported NIR-II dyes consist of the donor-acceptor-donor (D-A-D), and the HOMO is located along the whole molecular backbone whereas the LUMO is almost placed on the acceptor. The dominant transition from the ground to excited state (S_0_–S_1_) can thus be assigned as intramolecular charge transfer (ICT) from the HOMO to the LUMO. On the basis of each optical spectrum, the optical bandgap can be estimated as Equation (2) [89,90,91]:

(2) $$E_{\mathrm{opt}-\mathrm{gap}}(\mathrm{eV})=h\times f=h\times \frac{c}{\lambda_{\mathrm{abs}.\mathrm{edg}}}$$

where $E_{\mathrm{opt}-\mathrm{gap}}$ represents the optical bandgap (expressed in electron volts) and $\lambda_{\mathrm{abs}.\mathrm{edg}}\;$denotes the absorption edge wavelength derived from the strongest absorption wavelength, as schematically shown in Figure S1.

**Figure 4:**
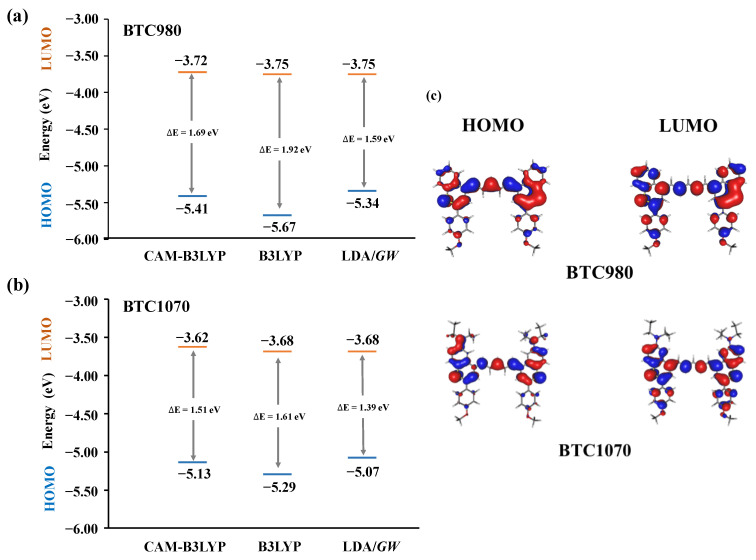
The HOMO and LUMO energy levels, and HOMO-LUMO gaps in (**a**) BTC980 and (**b**) BTC1070, as estimated using LDA/*GW*, B3LYP, and CAM-B3LYP calculations. (**c**) The HOMO, LUMO orbital distribution in BTC980 and BTC1070. The LUMO levels are shown in green, whereas the HOMO levels are shown in red.

Figure 4 predicts the frontier molecular orbitals of the HOMO and LUMO, along with the corresponding energy level of each structure. In the formation of D-A-D fluorophores, donor units are coupled with acceptor units to afford high fluorescence performance with a small HOMO-LUMO gap and a high absorption wavelength. Figure 4c shows that all of the density of the HOMOs were located along the π-conjugated backbone, which was higher than that of the LUMOs in both BTC980 and BTC1070 structures. With the added dimethylamino donor unit, the BTC1070 obtained a higher energy level than the BTC980 because of the contribution of N atoms in both the HOMO and LUMO of the BTC1070. On the basis of the calculation results shown in Table 1, the HOMO-LUMO energy gaps of the D-A-D NIR-II structures elucidated in both the *GW*-BSE and B3LYP approaches decreased in the order BTC980 > BTC1070, which is in total agreement with the corresponding reverse order of the longest absorption wavelength. The HOMO-LUMO gap values obtained from the *GW*-BSE calculations for all compounds were closer to the experimental excitation energy gaps than those obtained using either the B3LYP or the CAM-B3LYP functional.

## 4. Conclusions

We evaluated the electronic and optical properties of two different NIR-II fluorophores—including BTC980 and BTC1070—using the MBPT method within *GW*-BSE, standard (B3LYP), and long-range corrected hybrid function (CAM-B3LYP) calculations. Our implementation of the *GW* approximation enabled highly accurate estimates of the *GW* approximation efficiency. All B3LYP, CAM-B3LYP, and *GW*-BSE calculations showed S_0_–S_1_ excitation energies from the HOMO to the LUMO that verified the absorption wavelength trend of all two structures, which was described as a red shift of the strongest absorption wavelength in the order BTC980 < BTC1070. Moreover, the *GW*-BSE-computed vertical absorption wavelength and excitation energy levels were in remarkable agreement with the experimental wavelength and energy levels, even with respect to the shape of the absorption spectra in a vacuum and even in a solvent. A space-dependent Coulomb attenuation parameter involved in the *GW*-BSE can highly improve the accuracy of hybrid functional calculations in determining the optical and electronic properties of NIR-II fluorophores. In the present study, we emphasized that the BSE formalism relies on a many-body Green’s function formalism (*GW*-BSE) within the LDA of DFT, which enables a benchmarking approach to conduct calculations of the excited states of fluorophores.

## Figures and Tables

**Table 1:** Calculated maximum absorption wavelength ($\lambda_{\mathrm{abs}.\max}$), emission wavelength ($\lambda_{\mathrm{fl}.\;\max}$), and electronic transition composition for all structures computed in GW-BSE, B3LYP calculations, and the comparison with the experimental references.

Dye		$\lambda_{\mathrm{abs}.\max}\;$ (nm)	$\lambda_{\mathrm{fl}.\max}\;$ (nm)	$\lambda_{\mathrm{abs}.\mathrm{edg}}\;$ (nm)	$E_{\mathrm{opt}-\mathrm{gap}\;}$ (eV)	$E_{\mathrm{HOMO}-\mathrm{LUMO}\;}$ (eV)
	*GW*/BSE	923.4	975.1	1055	1.18	1.59
BTC980	B3LYP	921.8	931.1	990	1.25	1.92
	CAM-B3LYP	945	997	1098	1.13	1.69
	Exp [19]	932	980	995	1.25	
	*GW*-BSE	1014.7	1065.4	1175	1.05	1.39
BTC1070	B3LYP	1008.7	1016.8	1190	1.04	1.61
	CAM-B3LYP	1019.8	1059.2	1325.1	0.93	1.51
	Exp [19]	1014	1064	1156	1.07	

## Supplementary Materials

The following are available online at https://www.mdpi.com/article/10.3390/nano11092293/s1, Figure S1: Schematic representation of the UV absorption spectrum.
