# Supplementary material for: Optical and Electronic Properties of Organic NIR-II Fluorophores by Time-Dependent Density Functional Theory and Many-Body Perturbation Theory: GW-BSE Approaches
Source: Nanomaterials (Basel). 2021 Sep 3;11(9):2293. doi: 10.3390/nano11092293 (PMC8466807; doi:10.3390/nano11092293)
Supplement: Supplementary file 1 [file nanomaterials-11-02293-s001.zip › nanomaterials-1345598-supplementary.pdf]

Supplementary Materials

Optical and Electronic Properties of Organic NIR-II Fluorophores by Time-Dependent Density Functional Theory and Many-Body Perturbation Theory: *GW*-BSE Approaches

Nguyet N. T. Pham [1], Seong Hun Han [1], Jong S. Park [1,2,*] and Seung Geol Lee [1,2,*]

1 School of Chemical Engineering, Pusan National University, Busan 46241, Republic of Korea

2 Department of Organic Material Science and Engineering, Pusan National University, Busan 46241, Republic of Korea

* Correspondence: seunggeol.lee@pusan.ac.kr (S.G.L.) and jongpark@pusan.ac.kr (J.S.P.)

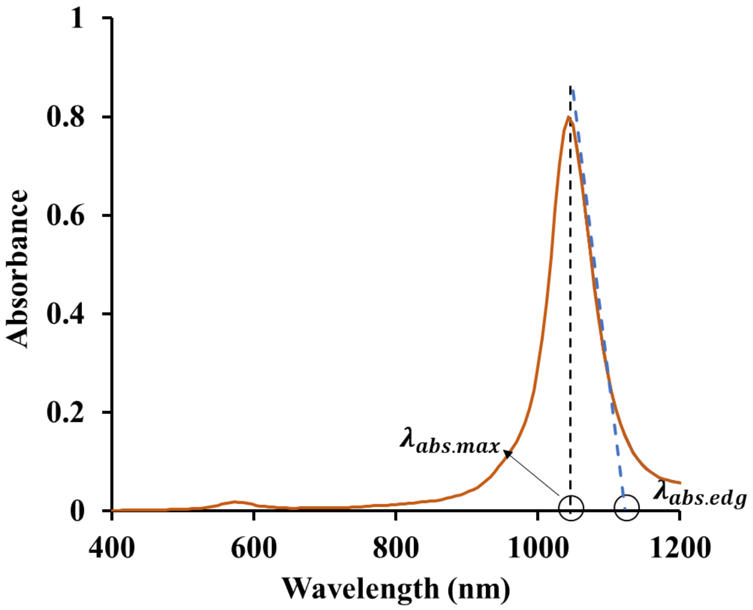

Figure S1. Schematic representation of the UV absorption spectrum.
